# 

**Published:** 1881-09-20

**Authors:** 


Entered at the Post-Office at Atlanta as second-class matter.

VOL. XI. SEPTEMBER 20, 1881. NO. 9

THE

### SOUTHERN MEDICAL RECORD:

A MONTHLY JOURNAL OF PRACTICAL MEDICINE.

Terms: $2 00 per Annum, in Advance; 4 Copies $6.00; Single Copies 20 Cents.

*"QUICQUID PRÆCIPIES ESTO BREVIS."*

### WHERE TO BUY.

William R. Warner & Co. .......... 1
Reed & Carnrick .......... 2-3
J. L. Berg & Co. .......... 4-5
University of Louisiana .......... 6
A. A. Mellier .......... 7
Pemberton, Pullum & Co. .......... 8
Mellin's Food .......... 9
Johnston's Fluid Beef .......... 9
Thomas F. Goode .......... 10
O. C. St. Clair .......... 11
Aloe & Hernstein .......... 12
Bedford Springs .......... 13
Schumann .......... 14
Powers & Weightman, .......... 14
J. A. Ansley .......... 14
N. Y. Pharmacal Association .......... 15
Geo. J. Howard & Bro .......... 16
Trommer Extract of Malt (ins. front cov.)
Reed & Carnrick ..........(inside back cover.)
Parke, Davis & Co. ..........(outside back cover.)
McKesson & Robbins .......... (colored leaves.)
Parke, Davis & Co. ..........(colored leaves.)
Scott's Emulsion ..........(colored leaves.)
Listerine ..........(colored leaves.)
Celerina ..........(colored leaves.)
Jefferson Med. College, Phila. (col. leaves.)
Southern Medical College ..........(col. leaves.)

Address R. C. WORD, M.D., Managing Editor, Atlanta, Ga.

